# A process evaluation of an on-line fall prevention and management program for individuals who use wheelchairs or scooters living with multiple sclerosis

**DOI:** 10.3389/fpubh.2022.1042668

**Published:** 2022-12-12

**Authors:** Toni Van Denend, Elizabeth W. Peterson, Amy Roder McArthur, Rebecca Yarnot, Jacqueline Kish, Sydney Steinkellner, Arman Sandhu, Laura A. Rice

**Affiliations:** ^1^Department of Occupational Therapy, University of Illinois Chicago, Chicago, IL, United States; ^2^Department of Disability and Human Development, College of Applied Health Sciences, University of Illinois Chicago, Chicago, IL, United States; ^3^Department of Kinesiology and Community Health, University of Illinois Urbana-Champaign, Urbana, IL, United States

**Keywords:** fall management, multiple sclerosis, wheelchair users, scooter users, telerehabilitation, complex intervention evaluation, mechanisms of change, implementation

## Abstract

**Background:**

Falls and resulting injury are a significant concern for individuals living with multiple sclerosis (MS) that use a wheelchair and/or scooter to support mobility. Effective fall prevention efforts are vital to support the health, wellbeing, and participation for these individuals.

**Aims:**

This study reports the findings from the process evaluation conducted in association with a pilot study evaluating the efficacy of Individualized Reduction of FaLLs-Online (iROLL-O), an online, group fall prevention, and management program specifically designed for community-based people living with multiple sclerosis (pwMS) who are full-time wheelchair or scooter users.

**Methods:**

A mixed-methods process evaluation was conducted, with specific attention to the impact of online delivery on intervention implementation, participant satisfaction, and mechanisms of change (MOC). Multiple data sources were utilized, including post-session and post-intervention participant and trainer feedback forms and participant qualitative interview data. Descriptive analysis was conducted using Microsoft Excel. Close-ended questions were analyzed by examining five-point Likert scale responses. Qualitative interview data was explored using thematic analysis.

**Results:**

Twelve participants and three trainers (one occupational therapist and two physical therapists) contributed to the study. Online delivery did not compromise session fidelity, which averaged 95%. No significant adaptations to the intervention were made during delivery. Participant satisfaction was high at 4.6/5.0. *Post-course Trainer Feedback Forms* indicate trainer satisfaction with the group dynamic, ability to address unique group needs, and program content. Reach improved with online delivery as transportation barriers were removed and recruitment from a broader geographic area was enabled. Three themes reflecting key MOC emerged from the analysis: group context, motivation for participant engagement, and the multifaceted nature of the program. The COVID-19 pandemic was identified as a contextual factor impacting community participation. Both participants and trainers identified the group dynamic as a strength. The trainers valued the program's flexibility in allowing them to address individual and/or group-specific fall prevention needs.

**Conclusion:**

Feedback from key stakeholders was essential to a meaningful process evaluation. Online delivery supported program implementation, including reach, and resulted in high levels of satisfaction among participants and trainers. Future iterations should aim to uphold the positive group context, recruit, and train skilled interventionists who are licensed as occupational or physical therapists and continue to provide the program's diverse approach to fall prevention and management.

## Introduction

Fall prevention is a recognized public health priority and falls pose a significant threat to the health and wellbeing of people living with multiple sclerosis (pwMS). Emerging evidence points to the high prevalence of falls and fear of falling among the 250,000 pwMS who use wheelchairs and scooters as their primary means of mobility and highlights the unique fall prevention needs of this population (1). Among wheelchair and scooter users living with multiple sclerosis (MS), between 50 and 75% report falling at least one time in a period of 6 months (2, 3) and multiple falls and fall-related injuries are common (2). Rice et al. (2) found that 76.7% of wheelchair and scooters users with MS reported concerns about falling and 65.9% limited their activities due to these concerns. Activity limitation associated with fear of falling may lead to deconditioning and ultimately a greater risk for falls (4). Activity limitation also has the potential to compromise quality of life and community participation (4, 5). Understanding the etiology and circumstances of falls among full-time wheelchair and scooter users is essential to informing intervention priorities. Studies involving non-ambulatory pwMS, i.e., pwMS who are unable to perform a timed walk test (6), indicate that falls in this population frequently occur during unavoidable routine activities, such as transferring and walking short distances (7). In addition to risk factors stemming from MS (e.g., compromised balance, weakness), pwMS experience behavioral, environmental, and psychological risk factors (e.g., fear of falling) that increase their risk of falling (8). Simply put, theses diverse and interacting influences lead to a high risk of falls and an imperative need for fall prevention and management programming.

In recent years important advances in evidence-based fall prevention and management programs designed for pwMS who use wheelchairs and scooters as their primary form of mobility have been made. Specifically, a single session, 45-min intervention created by Rice et al. (9) resulted in improvements in transfer quality, postural control, and reduction in fall frequency. Building upon that success, Rice et al. (10) created the Individualized Reduction of FaLLs-In Person (iROLL-IP) program. Individualized Reduction of FaLLs-In Person is a six-session, community-based intervention for full-time wheelchair and scooter users. It is delivered in-person to small groups of participants of approximately two to five people. The primary aim of iROLL-IP is to reduce fall incidence among full-time wheelchair and scooter users with MS. Secondary aims of the intervention are to improve functional mobility skills associated with fall risk (e.g., transfer and wheelchair/scooter skills, balance), increase knowledge of fall risk factors, decrease fear of falling, and enhance quality of life and community participation (10). Descriptions of the study undertaken to evaluate the feasibility and efficacy of iROLL-IP and the intervention itself, along with the complete iROLL-IP study protocol and participant manual, is described elsewhere (10). Briefly, the intervention is delivered by licensed physical and occupational therapists (herein referred to as “trainers”) and applies the health belief model (11), as well as Bandura's social cognitive theory (12) and features content to build participants' self-management of chronic fall risk. Development of the self-management content was informed by Lorig and Holman's operationalization of self-management, which includes six specific self-management skills: problem solving, decision making, resource utilization, the formation of patient–provider partnership, action planning, and self-tailoring (13).

Individualized Reduction of FaLLs (iROLL) program content is evidence-based (10). Specifically, the developers of the intervention drew extensively from their research to identify and address circumstances of falls among individuals who use a wheelchair or scooter (e.g., MS symptoms, environmental hazards, activity curtailment associated with fear of falling) (2, 7, 9, 14). Individualized Reduction of FaLLs-In Person includes concise didactic instruction on these topics, as well as exercises to improve sitting balance and core strength, and content designed to build wheelchair/scooter and transfer skills. Post-fall recovery and use of assistive technologies is highlighted in the intervention (15). Individualized Reduction of FaLLs trainers use a variety of educational strategies to actively engage participants ranging from group discussions, physical demonstrations, and practice opportunities. A number of resources support program implementation and receipt, including a program manual, videos, and pictures.

Skills built through the intervention range from developing transfer and wheelchair/scooter skills, building postural control and developing exercise habits to managing environmental hazards, completing wheelchair maintenance checks, and developing post-fall recovery plans (10). Participants are offered structured opportunities to incorporate their self-management skills into their lifestyle (10). These self-management skills take on many forms for participants depending on their individual needs. Some examples include navigating relationships with healthcare providers, and action planning.

Findings from the multi-site clinical trial undertaken to evaluate the efficacy of the iROLL-IP intervention demonstrated that 3 months after completion of the intervention, iROLL-IP participants demonstrated significant improvements in knowledge of fall prevention strategies (*p* = 0.01), knowledge of fall management strategies (*p* = 0.01), community participation in activities important to the participant (*p* ≤ 0.01), and transfer quality (*p* = 0.002). No significant differences were seen related to fall frequency, quality of life, seated postural control, or wheelchair skills (15). Process evaluation findings indicated iROLL-IP was implemented with high fidelity and that participants were highly satisfied with the intervention. Key mechanisms of change (MOC) ascertained through qualitative analysis of data yielded through trainer interviews included the group context, a strong program informed by evidence and interprofessional perspectives, and skilled interventionists (16). Online delivery was identified as a potential strategy to improve recruitment, which was identified as the most significant challenge associated with the iROLL-IP intervention.

These informative process evaluation findings, combined with the social distancing measures associated with COVID-19 infection control, led the research team to translate iROLL-IP to an online course: Individualized Reduction of FaLLs-Online (iROLL-O). Individualized Reduction of FaLLs-Online mirrors iROLL-IP in content, processes, and outcomes sought. Highlighted changes include adding asynchronous learning activities, trainer discussion guides, and the use of synchronous group videoconferencing. Like iROLL-IP, iROLL-O is a complex, group-based intervention with multiple potentially active elements that support change and/or impact key study outcomes.

This study reports the findings from the process evaluation (17, 18) conducted in association with the pilot study evaluating the efficacy of iROLL-O (19). The primary questions for this evaluation were: (1) How does online delivery impact intervention implementation (i.e., fidelity, dose, adaptations, and reach)? (2) Are participants and trainers satisfied with iROLL-O? (3) What are the key MOC associated with iROLL-O? Additionally, the process evaluation was intended to identify strengths and limitations of iROLL-O to inform the improvement of future delivery of the intervention.

## Methods

### Translation of iROLL-IP for online delivery

**Figure 1:**
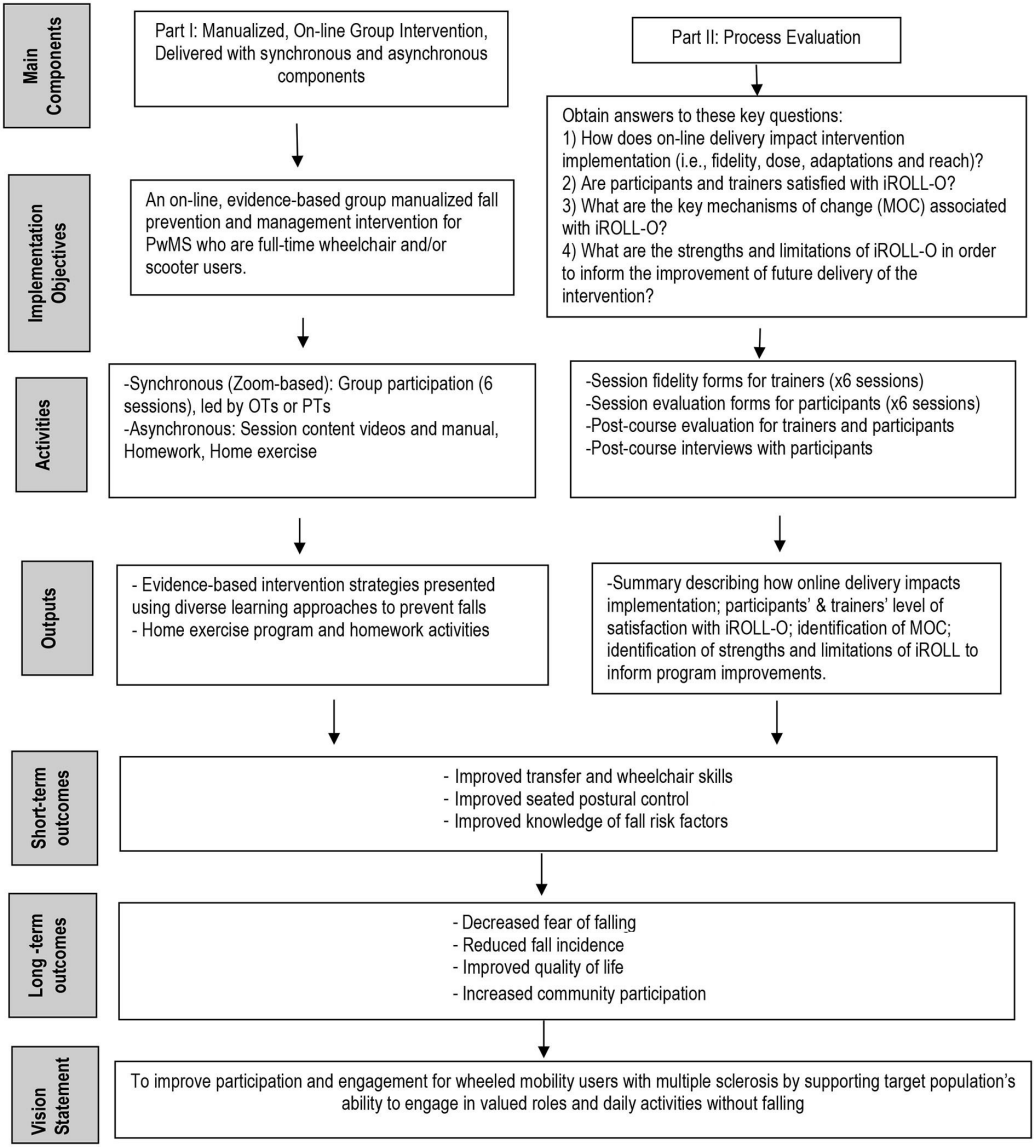
The iROLL-O intervention and process evaluation logic model.

In April 2020, iROLL-IP was translated to iROLL-O. Individualized Reduction of FaLLs-Online offered the same content as iROLL-IP and was created with the same desired outcomes in mind. Any adaptations made to translate iROLL-IP for online delivery were made with the intent of keeping key MOC identified through the iROLL-IP process evaluation intact. Specifically, the team sought to preserve the group dynamic, comprehensive nature of the program, strong program development, role of a skilled interventionist, and motivated participants (16). The logic model for iROLL-O and the present process evaluation is provided in Figure 1.

A series of adaptations were made to allow for remote delivery of the intervention. To begin, participants were required to have access to the Internet. The most substantial change was an increase in the use of asynchronous content. Specifically, in addition to the homework activities that were included in iROLL-IP (e.g., goal setting, journaling, exercise program, action planning) each week of iROLL-O, participants were asked to watch brief, pre-recorded videos in advance to the synchronous group time. During iROLL-IP, the trainers provided the program to study participants in six weekly, 2-h long, in-person, synchronous sessions. Between the weekly group sessions, participants were asked to set goals, write journal entries, and practice skills learned during the session. For iROLL-O, the weekly content was delivered online using asynchronous and synchronous learning strategies. A table describing key synchronous and asynchronous activities in each iROLL-O session is provided in Table 1. In line with recommended best practices for teaching online (20), the didactic presentations included in iROLL-O were brief (i.e., 5–7 min) each, adding up to approximately 60 min of content. Individualized Reduction of FaLLs-Online participants were also asked to set goals, write journal entries, and practice skills learned during the session. The planned dose of each synchronous session was 60 min per session. Compared to iROLL-IP, iROLL-O involved less time to practice functional mobility skills however detailed discussions about functional mobility technique and safety were retained.

**Table 1:** Synchronous and asynchronous session components for iROLL-O.

**Description of iROLL-O components**		
**Session no**.	**Asynchronous session components completed by iROLL-O participants in advance of synchronous session**	**Synchronous session components completed by iROLL-O trainers**
**Optional Session 0**	• Download Zoom	• Conduct Zoom session to ensure access to program technologies
**Session 1**	**Watch short video containing content that addresses the following objectives:**	
	• Introduce participants to the problem of falls and fall risk factors specific to wheelchair and scooter users with MS, including fear of falling. • Highlight the multifactorial nature of most falls and the importance of a multifactorial approach to managing fall risk. • Justify the importance of developing fall prevention strategies that (a) meet unique needs; (b) address risk factors operating within, and outside the individual; and (c) support participation in valued home and community-based activities. • Introduce the concept of journaling to reflect on new knowledge learned and goal setting for continued improvement. • Introduce therapeutic exercise program focused on enhancement of postural control and practice exercises. • **Complete:** fear of falling reflection, journal activity related to reducing fall risk, goal setting related to exercise, exercise program participation.	• Introduce participants to the program with an emphasis on program goals and the importance of group members sharing expertise/supporting each other during the six sessions. • Highlight that the program builds upon participants' strengths and expertise. The program activities were developed with the understanding that participants have experience with transfers, wheelchair management, etc., and the activities are designed to help the refine those skills to improve safety, save energy, and use the body in a way that is efficient and prevents overuse injuries. • Discuss regarding “ground rules,” such as maintaining confidentiality and being respectful to co-participants. • Identify participants' motivation for joining iROLL and key outcomes sought. • Answer questions regarding the therapeutic exercise program and help participants make adjustments based on their specific needs.
**Session 2**	**Watch short video containing content that addresses the following objectives:**	
	• Participants will be provided tips to help them perform transfers in a manner that reduces the potential for falls and conserves energy. • Participants will be provided tips to improve performance of basic wheelchair skills to enhance safety. • Participants will learn about common environmental hazards in the community and how they can be avoided. • Direct participants how to practice the therapeutic exercise program, transfer, and wheelchair skills with a care partner. • **Complete:** transfer and wheelchair skills reflection, home safety check list, action planning related to environmental modification needs, goal setting related to wheelchair and/or transfer skills, exercise program participation.	• Discuss the therapeutic exercise program and help participants problem solve through challenges faced related to the exercise program. • Draw from participants' experiences to review common environmental hazards in the home and community. • Problem solve management of common environmental hazards. • Discuss transfer and wheelchair/scooter skill practice and discuss challenges faced.
**Session 3**	**Watch short video containing content that addresses the following objectives:**	
	• Introduce post-fall management skills. • Instruct participants in the development or refinement of individualized fall management plans. • Continued education on refinement of wheelchair/scooter skills necessary for active community engagement. • Continued instruction on how to practice the therapeutic exercise program, transfer, and wheelchair skills with a care partner. • **Complete:** fall experience reflection, fall management plan worksheet, goal setting related to wheelchair and/or transfer skills practice, exercise program participation.	• Discuss importance of post-fall management. • Discuss development of individualized fall management plan and provide feedback on plan development. • Discuss challenges participants face in the community related to wheelchair skills.
**Session 4**	**Watch short video containing content that addresses the following objectives:**	
	• Provide instruction on refinement of advanced wheelchair/scooter skills. • Provide instruction on refinement of complex transfer for active community engagement. • Instruct participants on key strategies to manage common MS symptoms than can increase fall risk: Example: fatigue management. • Direct participants to continue practicing the therapeutic exercise program, transfer, and wheelchair skills with a care partner. • **Complete:** symptom related fall risk reflection, complex transfer reflection, and journal entry, participation-based goal setting activity, including an action plan, exercise program participation.	• Provide an opportunity for participants to ask questions about MS related symptoms. • Discuss the impact of skills learned through the iROLL program on confidence, quality of life, and community participation, and set realistic individualized goals for safe participation in home or community-based activities. • Discuss challenges participants face in the community related to wheelchair/scooter and complex transfer skills.
**Session 5**	**Watch short containing content that addresses the following objectives:**	
	• Educate users on different types of assistive technology to manage fall risk and how to access and maintain equipment. • Provide practice opportunities to perform and receive feedback on transfer and wheelchair skill techniques and the therapeutic exercise program. • **Complete:** fall reflection activity on wheelchair and scooter set-up, including action planning to minimize fall risk related to wheelchair or scooter set-up, journaling wheelchair and/or scooter related problems, wheelchair/scooter maintenance plan development, identify a wheelchair/scooter maintenance professional, exercise program participation.	• Discuss current assistive technology use and plans for obtainment of future technology. • Revisit impact of skills learned through the iROLL program on confidence, quality of life, and community participation. • Evaluate progress on goals for individualized activity.
**Session 6**	**Watch short video containing content that addresses the following objectives:**	
	• Educate users on methods to maintain skills and retain knowledge learned during the iROLL program. • Provide practice opportunities to perform and self-evaluate transfer and wheelchair skill techniques, exercise skills, and future needs. • **Complete:** iROLL skills learned activity, goal setting on iROLL skills maintenance, homework/practice activities, including goal setting, action planning, exercise participation.	• Provide a final opportunity for participants to ask clarifying questions about newly developed skills. • Compare strategies participants plan to use to sustain transfer, wheelchair, and exercise skills in order to prevent future falls.

Significant changes were also made to the trainer responsibilities and materials due to the reduced synchronous time. During iROLL-IP, the trainers presented didactic lectures, led discussions related to program material, and provided supervision as iROLL-IP participants practiced exercise, transfers, and wheelchair skills. During iROLL-O, the didactic content was presented during the asynchronous portion of the program. As a result, the trainers focused primarily on fostering discussion during the synchronous sessions. A detailed discussion guide was developed for the trainers to assure consistent discussion across each implementation of the program. Additional practice opportunities were offered. Participants were given the option of sending a video recording of a transfer and/or wheelchair/scooter skills and obtaining trainer feedback. Like the iROLL-IP training (16), the supplementary iROLL-O training was delivered by the project's Principal Investigator (LR) to all returning iROLL trainers.

### Study participants

As described by Rice et al. (10), the North American Research Committee on Multiple Sclerosis (NARCOMS) research registry was the primary vehicle used to recruit iROLL-O participants. All iROLL participants were ≥18 years old with a self-reported diagnosis of MS whose main form of mobility is a wheelchair or scooter. All reported the ability to perform transfers independently or required minimal to moderate assistance to perform transfers. All participants had experienced at least one fall in past 12 months.

### Trainers

All iROLL-O trainers were recruited by invitation only. All had experience delivering at least one cycle of iROLL-IP, were licensed as an occupational or physical therapist, had at least 2 years of clinical experience including at least 1 year of experience providing care to individuals with neurological impairments utilizing wheeled mobility devices, and had experience providing education to patients/clients in a group setting. As iROLL-IP trainers, iROLL-O trainers had previously participated in an iROLL-IP training workshop, which featured a thorough review of the facilitators' and participants' manuals. Strategies to maintain fidelity associated with both program content and delivery was described in detail. Individualized Reduction of FaLLs-In Person trainers also participated in ongoing meetings with the principal investigator to ensure questions were answered during the implementation of the intervention (16). Individualized Reduction of FaLLs-Online trainers received additional training to learn about iROLL-O processes, with an emphasis on program modifications associated with online delivery (e.g., use of the discussion guide instead of a trainer manual, revisions to the length of synchronous group time, inclusion of asynchronous content).

### Study protocol

Participants prospectively tracked fall frequency using a fall diary 12 weeks before and 24 weeks after the intervention. Outcomes were assessed pre, immediately post and 12-weeks post intervention regarding fear of falling, knowledge of fall prevention, mobility skills, quality of life, and community participation. Once assigned to a group and prior to each synchronous iROLL-O session, participants were asked to independently watch pre-recorded videos in specific content areas related to fall prevention and management. In addition to viewing asynchronous videos, iROLL-O participants were asked to complete an exercise program, goal setting, journaling, and action planning activities between sessions as homework. During the consent process the participants were informed that they were required to have assistance when engaging in any of the physical skills. In addition, during the synchronous sessions, the trainers reminded iROLL-O participants to practice physical skills with the help of another person. Trainers invited participants to report upon homework activities during synchronous group session. During the synchronous session, trainers facilitated discussions, highlighted key content-related messages for the week, and reviewed the exercise program. Participants were instructed to attend all six synchronous group sessions. Information describing key synchronous and asynchronous activities for each iROLL-O session is provided in Table 1. Participants received a $100 Amazon Gift Card for completing the study. The study protocol associated with the process evaluation is described below.

### Process evaluation data collection tools

Each iROLL-O participant was asked to complete a *Participant Post-Session Evaluation* after each iROLL-O synchronous session and a *Participant Final Course Evaluation Form* immediately following the sixth and final synchronous iROLL-O session. The forms included a mix of Likert scale satisfaction questions and open-ended feedback to ascertain perspectives on the program. Individualized Reduction of FaLLs-Online participants were also invited to participate in a one-on-one interview (audio only) led by a trained research assistant. Participant interviews were recorded and transcribed for data analysis purposes.

Each trainer was asked to complete a *Trainer Fidelity Form* after each iROLL-O synchronous session, and a *Post-Course Trainer Feedback Form* at the end of each course. These forms included a mix of Likert scale questions, Yes/No completion responses to specific fidelity items, and open-ended feedback on program strengths, limitations, and improvement opportunities.

Data collection strategies are delineated in Table 2. All forms used to collect iROLL-O process evaluation data were developed by the research team and were slightly modified versions of the forms used in process evaluation of iROLL-IP (16). The process evaluation forms used in iROLL-IP were adapted from work by Finlayson et al. (21). Individualized Reduction of FaLLs-In Person process evaluation used a Post-Course Trainer Interview guide and associated transcripts to inform MOC, which the iROLL-O process evaluation did not utilize. The only other modification to data collection tools between iROLL-IP and iROLL-O's process evaluations were slight wording revisions to accommodate the change to an online environment.

**Table 2:** iROLL-O process evaluation data collection strategies.

**Data source**	**Key measurement area**	**Completed by**	**When completed**
*Trainer Fidelity Form*	Implementation: fidelity, synchronous dose; trainer satisfaction	Trainer	Post-session
*Adaptation Log*	Implementation: adaptations	Principal investigator	Received post-intervention
*Research Staff Log*	Implementation: reach	Research staff	Completed on an ongoing basis during the study period
Post-*Course Trainer Feedback Form*	Implementation: fidelity; trainer satisfaction; mechanism of change; environmental elements	Trainer	Post-course
*Post-course Participant Interview Transcripts*	Mechanism of change; environmental elements	iROLL participant	Post-course
*Participant Post-Session Evaluations*	Participant satisfaction; mechanism of change; environmental elements	iROLL participant	Post-session
*Participant Final Course Evaluations Forms*	Mechanism of change; environmental elements	iROLL participant	Post-course

Revised from Van Denend et al. (16).

#### Intervention implementation data collection

Trainers completed a *Trainer Fidelity Form* to assess **fidelity** at the end of each synchronous session. At the end of the course, Trainers completed the *Post-Course Trainer Feedback Form* to assess perceptions of implementation strengths and weaknesses. **Synchronous dosage** was determined by calculating the duration of each iROLL session based on start and end times documented by trainers on *Trainer Fidelity Forms*. **Asynchronous dosage**, including practice activity completion was not tracked for this study. **Adaptations** were identified via documentation and communication (i.e., *Adaptation Log*) with the PI (LR) of the study. **Reach** was tracked by a research staff member, who monitored interest calls, eligibility, and attendance (i.e., *Research Staff Log*). The staff member also monitored attrition of study participants by completing telephone calls with those missing sessions and maintaining findings on an internal tracking log.

#### Participant and trainer satisfaction data collection

Participant satisfaction data was collected using the *Participant Post-Session Evaluations* following each session. Trainers used the *Trainer Fidelity Form* after each session and the *Post-Course Trainer Feedback Forms* at the end of each course to rate their perspective on program features and satisfaction. Data was collected confidentially via REDcap and participants were e-mailed a link from the study coordinator to complete the assessments independently online. The trainers were not involved in the data collection process of the feedback forms.

#### MOC data collection

To examine MOC, relevant data were extracted from the *Participant Post-Session Evaluations, Post-course Participant Interview Transcripts, Participant Final Course Evaluations*, and the *Post-Course Trainer Feedback Forms*.

### Process evaluation data analysis

Descriptive analysis was conducted using Microsoft Excel (Redmond, WA). As with iROLL-IP process evaluation (16), close-ended questions were analyzed by examining five-point Likert scale responses. Open-ended survey response data was reviewed, categorized, and discussed by the investigative team. Qualitative interview data was explored using thematic analysis (22). Using a shared code book, two team members conducted independent open coding. The code book was later refined to address coding discrepancies and newly emerging codes. Initially, all four coders ensured intercoder reliability until consensus was reached for definitions of codes. After consensus was established, coding was performed in pairs for the remaining interview. To develop the code book, codes were grouped into themes, definitions developed and refined, and key representative participant quotes were identified for each code.

Using triangulation to support internal validity of findings, a member of the investigative team (TV) compared the findings that emerged from the transcripts of the telephone interviews conducted with iROLL-IP participants, to the *Participant Post-Session Evaluations, Participant Final Course Evaluations*, and the *Post-Course Trainer Feedback Forms*.

### Ethics

The study was approved by the Human Research Protection Offices at the three collaborating sites: the University of Illinois at Chicago (UIC), the University of Illinois Urbana-Champaign (UIUC), and the Shepherd Center (SC) in Atlanta, GA. All study participants provided informed consent prior to engaging in any research activities.

## Results

Results from three trainers and all available process evaluation data are presented herein, including participants who dropped out of the program at various points. Seven cycles of iROLL-O were delivered between June 2020 and May 2021. Trainers ran between one and four iROLL-O groups. When assigned a group, a trainer conducted each of the six sessions. Process evaluation data was collected between June 2020 and May 2021.

### Description of iROLL-O participants and trainers

Key characteristics of the 12 iROLL-O participants that completed follow up testing after the intervention can be found in Table 3. Of the 12 participants that completed all iROLL-O follow-up testing, six attended all six synchronous group sessions, five attended five sessions, and one attended four sessions. The majority of participants were women (92%), with an average age of 62 (SD ± 12) years and reported time with MS averaging 27 (SD ± 13) years. Participants reported using a wheelchair for an average of 11 (SD ± 4) years. The participants lived in nine different states; eleven lived in urban areas and one lived in a rural area. The three trainers involved in the present study had an average of 16 years in practice (SD ± 2 years) and included one occupational therapist and two physical therapists.

**Table 3:** Characteristics of the iROLL participants*.

**Variable**	Participants^*^
Age: years, [mean], (range)	62.33, [12.15], (38–76)
Gender [*n* (%)]	Female 11 (92%) Male 1 (8%)
Types of MS [*n* (%)]	Secondary progressive 6 (50%) Relapse-remitting 3 (25%) Primary progressive 2 (17%) Progressive relapsing 1 (8%)
Time with MS: years, [mean], (range)	26.58, [12.63], (4–50)
Years of current wheelchair use: years, [mean], (range)	10.91, [4.42], (3–16)
Primary mobility device use: hours per week, [mean], (range)	82.45, [46.38], (18–185)
Type of wheeled mobility device [*n* (%)]	Power W/C: 6 (50%) Manual W/C: 3 (25%) Scooter: 3 (25%)
Participant location	9 different states 11 from Urban areas^**^ 1 from a Rural area
Number of falls in the past 6 months: *N*, [Median], (range)	2.75, [2.67], (1–10)
Experience with Zoom or telehealth (Yes/No) (%)	7 Yes (58%) 4 No (33%) (1 missing)

^*^People living with MS who are full-time wheelchair or scooter users who completed all iROLL-O follow up testing.

^**^As identified by areas >5,000 by the Department of Commerce (23).

### Intervention implementation findings

**Fidelity** findings indicated that the intervention was delivered with 94.9% fidelity, on average. The lowest fidelity score was associated with Session 6 (89.3%) and the highest fidelity score was associated with Session 3 (97.4%). The items on the fidelity forms that primarily related to logistics (e.g., starting on time, providing a reminder about completing course evaluations) were scored the lowest by trainers. Content-related items (e.g., “I provided an overview of MS symptoms impact on fall risk”) were rated high by trainers, with the exception of one item ranked slightly lower than the others (i.e., Session 1 SMART goals at 71.4%).

Regarding **dose findings**, each iROLL-O session was designed to have 60 min of synchronous session activities. Session 3 was rated the longest, averaging 78 min and Session 5 was the shortest at 58 min. The average synchronous time for iROLL-O was 65 min, per reports from session trainers. No significant **adaptations** were made during the course of the intervention.

**Reach** findings indicated 18 individuals were deemed eligible and planned to participate in iROLL-O. Three withdrew prior to the intervention: one individual was deemed ineligible, one was lost to follow up, and another reported the intervention did not fit their needs. Three additional individuals dropped out after their iROLL-O cycle began: one was hospitalized, one reported an injured spouse who required care, and a third was lost to follow-up. Twelve individuals completed the program. The number of iROLL-O participants in each group ranged from two to three individuals. Table 4 summarizes fidelity scores, dose, and attendance; Table 5 summarizes iROLL-O's reach.

**Table 4:** Findings from iROLL-O fidelity forms.

**Session**	**Fidelity**	**Synchronous dose**
	**(No. of completed fidelity items/Total no. of fidelity items)**	**(Length of session in min)**
Session 1	95.24%	68
Session 2	94.81%	62
Session 3	97.40%	78
Session 4	96.10%	68
Session 5	96.43%	58
Session 6	89.29%	60
**Average**	**94.88%**	**65**

Bolded terms offset the average from each of the individual session scores.

**Table 5:** Summary of iROLL-O's reach.

	**iROLL-O**
Screened	32
Did not pass screening	12
Declined due to transportation/distance	0
Withdrew before visit 1	2
Enrolled iROLL participants	18
Withdrew prior to intervention (Ineligible = 1, Did not feel intervention fit needs = 1, Lost to follow up = 1)	3
Withdrew during the intervention or during follow up (Hospitalization due to MS = 1, Change living situation mid-intervention = 1; Lost to follow up = 1)	3
iROLL participants who completed all iROLL-O follow up testing	12

### How online delivery impacted implementation and participant experience

Many participants appreciated the accessibility of the online program. In fact, participation in a face-to-face program clearly would not have been an option for some iROLL-O participants.

- …*if it was in person I don't know that I could have participated because I don't know that I would have been able to get there* (Age 67, Scooter User).
- *Well, I think it was probably a little more adaptable, you can do a little at a time and turn it off if you wanted to and go back to it. I kinda like that idea rather than having to spend 2 h at a time. Maybe, it was easier the way we did it* (Age 73, Power Wheelchair User).

Despite the benefits of online delivery of the program, there were also some drawbacks. Notable challenges included technical problems and limitations with transfer practice as a result of being virtual.

- *Personally, I was not able to join in on the meeting because the computer took so long to power up and I was late* (Age 74, Power Wheelchair User).
- …*One seemed to have signed in and then walked away—never responded to questions and we only saw her ceiling* (Trainer Feedback).
- *Although virtual sessions are great and have some benefit they do limit the ability to practice or provide a lot of practical feedback on how people are able to transfer* (Trainer Feedback).
- …* it probably would have been better to learn in person. You know, instead of watching a video of someone getting up, actually being there and you know... showing me how to get up* (Age 38, Manual Wheelchair User).

### Participant and trainer satisfaction findings

Participant satisfaction based on findings from data yielded by the *Participant Post-Session Evaluations* was high (4.6/5.0). The highest satisfaction scores were associated with Session 5 (4.8/5.0) and the lowest scores were associated with Session 2 (4.5/5.0). Across all sessions, participants reported the highest satisfaction with the following program features: Week 4—Follow up home exercise training 4.9/5.0; Week 5—Follow up home exercise training 4.9/5.0; and Week 5—Knowledge of assistive technology to manage fall risk 4.8/5.0. Likewise, participants rated the following program features the lowest across all sessions: Week 4—Practice opportunities to refine wheelchair safety skills 4.3/5.0, Week 3—Training on complex transfers 4.3/5.0, and Week 2—Wheelchair/Scooter safety skills training 4.3/5.0. Tables 6, 7 outline the participant satisfaction reporting.

**Table 6:** Summary of iROLL-o's post-session participant satisfaction (on scale of 1–5: 1 = poor; 5 = excellent).

**Session**	**Average**
1	4.68
2	4.45
3	4.48
4	4.53
5	4.77
6	4.63
Average	4.59

**Table 7:** Summary of iROLL-O's post-course participant satisfaction (on scale of 1–5: 1 = poor; 5 = excellent).

**Item**	**Rating**
Instructor's knowledge of the course content	5.00
Instructor's ability to present course material and to facilitate discussion	4.93
Physical environment of the course allowed you to see, hear, concentrate, and participate	4.71
Overall value of the course content to help you manage falls	4.64
Ability of course and instructor to motivate you to try new fall prevention strategies	4.57
Overall quality of homework review sessions at the beginning of each session	4.50
Overall quality of the manual	4.43
Overall quality of homework	4.36
Format of the course	4.36
**Overall rating score**	4.61

Program features rated the highest by trainers, as indicated by the completed *Post-Course Trainer Feedback Forms*, were: participants were given enough time to engage in and benefit from social learning; the program supported participants' ability to manage several types of fall risk factors; and the program improved knowledge and management of fall risk factors (all 4.8/5.0). The three lowest rated program features were: the program improved community participation 3.6/5.0; the program improved participants' ability to manage wheelchair skills and transfers 4.2/5.0; and the quality of the manual with respect to its usefulness in supporting one's ability to facilitate and deliver the iROLL intervention 4.2/5.0. The most essential program features as reported by trainers included: the videos, group discussion/context, transfer skills, and wheelchair skills and maintenance. The least essential program features included wheelchair or scooter higher-level skills (e.g., navigating curbs and ramps), and community participation discussions.

### MOC findings

The *Post-course Participant Interview Transcripts* were the primary data source yielding insights into MOC, with data from *the Post-Course Trainer Feedback Forms, Participant Post-Session Evaluations*, and *Participant Final Course Evaluations* adding additional insights. Three major themes emerged: group context, motivation for participant engagement in iROLL-O, and the multifaceted nature of the program.

#### Theme 1: The group context

Participants highlighted the value of the group context, which allowed them to encourage each other and share problems, ideas, experiences, and information. The trainer was often mentioned as a positive influence on the group discussion.

- “*It's nice to have a small group of people that you'll get to know and will share your problems”* (Age 60, Power Wheelchair User).
- “*In my group we share learned experiences, along with encouraging suggestions with each other. Our facilitator is great, she allows for open discussion without compromising the module info for the week”* (Age 74, Power Wheelchair User).

#### Theme 2: Motivation for participant engagement in iROLL-O

The participants described their intrinsic motivation to participate in iROLL-O. Participants' reasons for joining the iROLL-O program ranged from recognizing a history of falls and a desire to learn skills to prevent or manage falls to contributing to research, receiving financial compensation, and accessing social support:

- *I have lots of falls in my history because no one actually gave me a license to use this [wheelchair], you know… I just made a terrible mess and no one gave me a license so that explains it* (Age 73, Power Wheelchair User).
- *I wanted to learn how not to fall and different ways to use my wheelchair because I was never taught and I was just subscribed hey I think you need to be in a wheelchair since I didn't go to any therapy or anything. You know I didn't learn how to get up from a fall or what to do when you go over a bump or anything like that so I was really interested in what the program could offer* (Age 38, Manual Wheelchair User).

#### Theme 3: Multifaceted nature of the program

Trainers and participants alike spoke to the benefit of the wide range of topics covered in iROLL-O and the diverse learning approaches used. The intervention's ability to support both current and future fall prevention needs and its attention to fall management was specifically highlighted, as was the value of the content on fear of falling, fall management, self-management, self-awareness, environment-related safety considerations, and transfer training. Both trainers and participants described the usefulness of the resource materials, exercise, and the videos.

- …* I have become more aware, like if you're rushing … I can't be rushing* (Age 73, Power Wheelchair User).
- …* I know that when I have to transfer from the wheelchair to a chair or from the wheelchair to the toilet or to the shower, I need to make sure that there's really a clear access path... the program was great and made me aware and made me start doing it* (Age 59, Manual Wheelchair).
- *I think it was a lot of help cause it gave me different views of doing different transfers and well it did as we said it opened my eyes to how I could be my own worst enemy when it comes to falls. Well I was just like going through life thinking that I can do this no problem and being sorta nonchalant about things and falling* (Age 67, Scooter User).
- *This course was very informative. I feel like my core has gotten stronger from exercises. Transfers are still pretty difficult for me being in a scooter so this course has taught me that I need to investigate getting a power chair…* (Age 62, Scooter User).
- *And It was helpful in a lot of ways and it gave me and idea of what to prepare myself for in the future because I am probably headed toward not being able to use a scooter anymore or having to put a lift in my van and stuff like that, where I haven't had to do that yet so far* (Age 64, Scooter User).

### Contextual factors

The present study occurred during COVID-19 pandemic. Data from the *Post-course Participant Interview Transcripts, Participant Post-Session Evaluations, Participant Final Course Evaluations*, and the *Post-Course Trainer Feedback Forms* provided important insights into the influence of COVID-19 on the participants' experience of the intervention. Individualized Reduction of FaLLs participants described the negative impact of the pandemic on their activity levels:

- …*I haven't been doing anything* (Age 64, Scooter User).
- *Because I really missed being able to go to a gym and do any workout, so this helps me* (Age 76, Manual Wheelchair).
- *COVID-19 has put a halt to any attempts to get engaged in community activities* (Age 67, Power Wheelchair).

Likewise, trainers identified the pandemic as the probable reason that conversations about community participation and engagement were so challenging:

- *It was difficult to engage participants in discussion of community participation. Not sure if is due to COVID-19 restrictions or lack of participation in other parts of the program* (Trainer Feedback).
- *Participants did not have much to say about community engagement as they are not really accessing the community due to COVID. It made this topic of discussion somewhat difficult and irrelevant* (Trainer Feedback).

Winter weather was another contextual factor that negatively impacted participation in activities outside of the house. Participants who engaged in iROLL-O during the winter months clearly curtailed activity due to dangers posed by snow and ice. One participant stated, “*I am a ‘victim' of snow and ice which makes me homebound”* and another, “*…Every time I have a plan [to buy new DME] the weather changes so I don't want to go out and experience the life of falling on the snow or ice…”* (Age 67, Power Wheelchair User).

The final contextual factor identified was living in areas where access to physical or occupational therapy was limited, which negatively impacted participants' ability to pursue recommended services upon the conclusion of iROLL-O. One participant stated, “…*the suggestion is to find a PT or OT that can show how to do the technique being demonstrated. I do not live in an area with those services easily accessed…”* (Age 59, Manual Wheelchair User).

### Key strengths, limitations, and recommendations to improve iROLL-O

Findings from the *Trainer Fidelity Forms* highlighted many program strengths/facilitators and challenges/barriers (See Table 8). Trainers emphasized the value of the positive group dynamic, the quality videos and content, and the motivated participants as key strengths/facilitators for iROLL-O. They also valued the program's flexibility in allowing them to address individual and/or group-specific fall prevention needs. With respect to challenges or barriers, trainers noted that the “relatability” of some of the videos or manual content could be improved, especially for scooter users. The trainers offered suggestions to improve the exercises, and to more effectively manage session time and technological issues. Suggestions on supporting community participation during the COVID-19 pandemic were also noted.

**Table 8:** Summary of iROLL-O's *Trainer Fidelity Form* open ended coding.

**Facilitators/Positives (*N* = number of responses for category)**	
Positive group dynamic (Participant resource sharing)	10
Ability to “individualize” or increase focus on group selected material/topics (GB, backward falls, bathroom transfers, fall alert device, w/c fittings) Note: includes flexible trainer decision making/addressing group individualization needs	7
Quality videos or content (promoting safety awareness/helpful with early access pre-session)	4
Motivated participants	3
**Barriers/Challenges (*****N*** = **number of responses for category)**	
Relatability of or additional videos and images in manual (notes related to scooters; directing CGs, outdoor curb navigation)	13
Time issues: late start/late end/extra time needed (more time needed on proper body positioning and biomechanics, late start d/t tech challenge, late joiner to group)	8
Exercise issues (Declining to practice exercises, difficulty with scooting exercise, exercise tracking, complicated professional jargon used)	8
Technology challenges	4
Community participation challenges due to COVID-19	4
Long-term maintenance concerns/Follow-up requested	3
Availability of resources	2
Unengaged participant	1
Only one attending group	1
None or general “went well”	23

Participants and trainers also highlighted specific areas for program improvements. For example, one participant stated, “*The manual could use a bit of visual break between sections … the sections visually run together. A header or banner could be added to identify Sections. Maybe a few asterisks at the edge of a section page would help break up the run-on appearance of the content”* (Age 65, Scooter User). Trainers also requested a hard copy of the participant manual.

Some participants found the selected images, models, information, and transfer samples unrelatable to their experience.

- *I think the real challenge, besides the type of wheelchair that I had that some of the things I wasn't able to do, also seeing like the demonstrations, they weren't necessarily for me. It was like maybe if they had someone that had more difficulty being able to get up. Maybe having someone with my type of MS instead of just saying “hey just put your leg up here” and doing it like its nothing because its not nothing* (Age 38, Manual Wheelchair User).

Participants provided suggestions to specific aspects of the program, including improving the wheelchair maintenance and exercise portions of the program. One participant reported, “*Well, maybe I would say that the only part that I didn't think was wonderful was the maintenance and the back and forth of how we were supposed to do such and such every month or every 6 months and back to something that was once a year and you know it was sort of erratic…”* (Age 73, Power Wheelchair User). Others had suggestions to improve the exercise portion of the program, such as simpler exercise naming, adding more leg exercises, and the potential of a separate, optional exercise meeting.

Finally, trainers and participants agreed on the need for more practice opportunities, especially to hone, wheelchair and transfer skills, as well as a preliminary session occurring before Session 1 to acquaint the group with the technology associated with the mode of delivery.

- *I think the online version is great—it expands the program to many more people and decreases demands on transportation. But I think more can be done to incorporate “doing” into the sessions—planning to “do” a transfer during the session by creating a plan to have a caregiver present or planning to “do” w/c skills by signing in on your phone that day…* (Trainer Feedback).

Table 9 provides a full summary of strengths, limitations, and associated recommendations of iROLL-O.

**Table 9:** Summary of iROLL-O's strengths, limitations, and recommendation.

**Key strengths**	**Source (s)**	**Recommendations**
**Description of strength**	
Online experience	*Participant Final Course Evaluation, Trainer Final Course Feedback Form Participant Post-Session Feedback Form, Post-course Participant Interview Transcripts*	Outside of a few technical challenges, the online forum was a satisfactory and feasible means to deliver the synchronous portion of the course. Recommend ongoing utilization of online delivery.
Implementation: Quality trainer training (Rated 5.0/5.0 by Trainers)	*Post-Course Trainer Feedback Form*	Maintain the current training approach.
Implementation: Delivered with high fidelity (Rated at 94.88%)	*Trainer Fidelity Form*	Adequate training and manualized material appear to be supporting fidelity. Maintain these efforts.
Implementation: Asynchronous video utilization	*Trainer Final Course Feedback Form, Participant Post-Session Feedback Form*	Using videos prior to the session appeared satisfactory to participants and trainers, although minor revisions are suggested to the content in the MOC recommendations.
MOC: The group context	*Trainer Final Course Feedback Form, Trainer Fidelity Form, Participant Post-Session Feedback Form, Participant Final Course Evaluation, Post-course Participant Interview Transcripts*	Continue to ensure the utilization of a group and skilled trainers in future iterations.
MOC: Motivated participants	*Trainer Fidelity Form, Participant Post-Session Feedback Form, Participant Final Course Evaluation, Post-course Participant Interview Transcripts*	Recruitment efforts and program topic area are of interest to participants. Continue to consider expansion of recruitment to include integration of key participant motivators for future recruitment efforts.
MOC: Multifaceted nature of the program	*Trainer Final Course Feedback Form, Trainer Fidelity Form, Participant Post-Session Feedback Form, Participant Final Course Evaluation, Post-course Participant Interview Transcripts*	Participant and trainers alike are satisfied with the scope of fall prevention and management areas covered in the program. Continue to offer these diverse areas, including the utilization of varied learning approaches (didactic teaching, group discussion, manuals, asynchronous videos, and practice activities).
**Key limitations**	
**Description of limitation**	
Implementation: Manual formatting (availability of hard copies, general formatting, Trainer manual revisions)	*Trainer Final Course Feedback Form, Participant Post-Session Feedback Form, Participant Final Course Evaluation*	Consider enhancements to the iROLL-O trainer material, making hard copies of the participant manuals available for trainers and consider investing in enhancements of the formatting (increase visual appeal, adding tabs) to participant manual.
Implementation: Recruitment	*Study Coordinator log*	Recruitment was slow. Using online delivery, consider expanding to additional states/areas. Will need to explore cross-state licensure for occupational and physical therapists to deliver an intervention across state lines. Alternatively, have cohorts arranged by state with licensed therapists in each state to deliver the intervention.
Implementation: Technical challenges (logging onto/fully accessing Zoom)	*Participant Post-Session Feedback Form*	As mentioned in the trainer feedback, consider a “Group 0,” where the only thing addressed is ensuring participants are both confident and able to access necessary technology. Training and a technical support team to on-board both trainers and participants to field necessary technology is recommended. Consider availability of loaned hot spots in poor internet serviced areas.
Implementation: Starting on-time	*Trainer Fidelity Form*	A “Group 0,” adequate onboarding and technical support will help with the issue of starting on time.
Environmental element: Challenging to impact community participation (due to COVID)	*Trainer Final Course Feedback Form, Trainer Fidelity Form, Participant Post-Session Feedback Form, Post-course Participant Interview Transcripts*	Consider additional training and material to support trainers in addressing participation challenges. COVID-19 mitigation efforts are lessening, but participation recovery may need additional support for various reasons (e.g., deconditioning, decrease social connectedness, fear).
Improvement Recommendation: Exercise issues (declining to practice, difficulty with scooting exercise, exercise tracking, complicated professional jargon, wanting additional time exercising)	*Trainer Fidelity Form, Participant Post-Session Feedback Form*	Regarding those declining to practice, one participant suggestion was to offer an additional (potentially optional) session that is geared toward exercise (form, accountability to engage in, etc.). Recommend further exploration of the lower extremity exercise included, as well as the scooting exercise as participants expressed suggestions in these areas. Recommend simplifying the exercise tracker and consider renaming exercises, using lay language.
Improvement Recommendation: Relatability of program material, content, or videos to each participant's current skill or functional level	*Trainer Final Course Feedback Form, Trainer Fidelity Form, Participant Post-Session Feedback Form, Participant Final Course Evaluation, Post-course Participant Interview Transcripts*	Some participants expressed interest in additional scooter related images, examples and content, in addition to video examples of what to do in more challenging transfer scenarios. Recommend consider expanding the representation of various disability levels and devices used in videos, images, examples, etc. As possible, use more natural environmental settings over more clinic-based settings.
Improvement Recommendation: Long-term maintenance	*Trainer Fidelity Form, Participant Post-Session Feedback Form*	Recommend engaging care partners in certain program sections (e.g., exercise and transfer training especially) to support long-term maintenance. Could consider a semi-regular group check-in built in monthly post-course to support long-term maintenance.
Improvement Recommendation: Declining to engage in practice activities or compromised practice opportunities d/t being online	*Participant Post-Session Feedback Form, Trainer Final Course Feedback Form*	One trainer suggested sending sample videos of participant transfers to provide feedback. Increasing care partner engagement in the practice portions of program may also increase practice opportunities.

## Discussion

The COVID-19 pandemic, combined with reach-related challenges identified in the process evaluation of iROLL-IP (16) created an imperative for this process evaluation conducted in association with a pilot study evaluating the efficacy of iROLL-O, an online fall prevention and management program for individuals living with MS who are full-time wheelchair or scooter users. The diverse data collection strategies utilized yielded answers to the key questions intended to be addressed through the process evaluation. Among these strategies was the plan to collect both quantitative and qualitative data from of two groups of stakeholders: end users and interventionists.

Findings demonstrated that online delivery resulted in high levels of satisfaction among participants and trainers. Participants were especially satisfied with the home exercise program and training on use of assistive technology to manage fall risk. Findings also demonstrated that online delivery supported program implementation, including program reach. It is evident that online delivery facilitated increased access to iROLL. In iROLL-IP, 32% of screened individuals declined to participate either due to transportation related issues or time/scheduling issues (16). Such barriers were not reported in iROLL-O. This study supports the findings of Banbury et al. (24) who reported that videoconference delivery may improve program accessibility, especially for those with limited mobility. Our findings also build the growing body of evidence demonstrating the value of online delivery formats to support healthy lifestyle behaviors for wheelchair users. For example, Hoevenaars et al. (25) studied wheelchair users with spinal cord injury or lower limb amputation and found that supporting physical activity, diet, sleep, and relaxation using a developed mobile application was feasible and led to high levels of participant satisfaction. Building upon the success of the online delivery, use of mobile application technology could be a consideration for future iterations of iROLL-O to support interactions among program participants, interactions among the program participants and the trainer, and long-term self-management of fall risk.

Positive group dynamics, the multifaceted nature of the program and the motivated participants were identified as key MOC supporting attainment of program outcomes. These MOC provided evidence that social learning theory and self-management strategies were effectively applied in iROLL-O. Trainers were notably satisfied with the group format, the social learning that occurred, and the program's ability to improve participants' self-management of diverse fall risk factors. Similar to the experience reported by Banbury et al. (24), effective group processes were maintained through online delivery. Our process evaluation findings clearly pointed to the value of involving licensed occupational and physical therapists as trainers. The iROLL-O trainers drew from knowledge and skills gained through their extensive work experience (16 years, on average) to facilitate the iROLL-O group process and individualize program content while maintaining program fidelity. The trainers' feedback regarding their high level of satisfaction with the training they received in advance of delivering iROLL-O was important given that administering healthcare services using telehealth requires adequate training to ensure competent delivery of quality services (26).

As intended, the process evaluation led to identification of several opportunities to improve iROLL-O. Participants and trainers alike supported the revision of program material to increase relatability to more diverse functional levels and devices utilized. For example, more images portraying scooter users were recommended. Importantly, the need for more opportunities for participants to practice wheelchair skills and transfers was identified through several sources. The challenges associated with teaching wheelchair skills via telehealth are not unique to iROLL-O. In an intervention aimed at evaluating an mHealth wheelchair skills program for older adults using manual wheelchairs, Giesbrecht and Miller (27) reported challenges improving wheelchair skills capacity but found positive results improving safety. Bell et al. (28) discovered the value of evaluating wheelchair skills and accessibility in a client's natural environment using telehealth but emphasized the value of telehealth services in partnership with traditional in-person services to support functional outcomes. Future iterations of iROLL-O could consider a hybrid approach that compliments online learning with in-person activities. A one-on-one in-home visit that provides participants with the opportunity to practice wheelchair and transfer skills in their natural home environment under the supervision of the iROLL-O trainer could be utilized. Alternatively, a group of iROLL-O participants could meet with the iROLL-O trainer together, in-person, to refine skills. Meeting as a group to work toward skill mastery would allow for peer modeling and may increase the potential for enhanced falls self-efficacy (29).

Additional areas for improvement identified through the process evaluation pertain to addressing challenges associated with online delivery. Future iterations of iROLL-O can utilize an iterative process to onboard participants and trainers, and develop a protocol for technical support. An optional pre-intervention session was offered to support/check technology and orient participants to the program, but few chose to attend. Rather than making this pre-intervention session optional, an iROLL-O “Session Zero” designed to orient participants to the online platform and support services available can integrated into the iROLL-O program to mitigate technology-related issues noted by participants and trainers. Finally, while expansion of delivery of the iROLL program using the online form is warranted, professional state laws and regulations related to telehealth need to be considered, especially when delivering services across state lines (30).

Understanding the context in which iROLL-O was delivered goes beyond consideration of online delivery. The iROLL-O cycles took place between June 2020 and May 2021, a period when social distancing was heavily utilized to mitigate risk of infection. The pandemic had a significant psychological impact on pwMS, including a “higher burden of depressive symptoms, a worse sleep quality and perceived an increase in fatigue level” compared to the general population (31). The pandemic also prompted a decrease in community participation for many, especially for those with mobility limitations (32). Thus, the process evaluation findings yielding insights into the COVID-19 pandemic's impact on the participants' experience of the iROLL-O intervention and outcomes sought are noteworthy. Two key COVID-19-related findings were identified through the process evaluation. First, compared to iROLL-IP trainers, iROLL-O trainers were less likely to report that community participation increased among the people living with MS who were participating in the study they were a part of. Second, in light of reduced opportunities for socialization, access to an online community was welcomed by many iROLL-O participants. For example, one participant stated, “…*things that you couldn't participate before because you couldn't get out, now everything is being held online, like church and meetings, like my MS meetings they're all zoom now and they got a MS zoom dance class they can be done in a chair, so yeah so zoom has been awesome really. I really like that and I really liked that part of the study*” (Age 62, Scooter User). Overall, the process evaluation findings provide an important reminder that study results related to community participation must consider contextual influences on the outcome.

### Limitations

There are several limitations associated with this study that require consideration. To enhance learning, trainers reviewed and highlighted key points of the asynchronous session content during the synchronous group sessions. However, asynchronous dosage received by the participants was not tracked. The research team cannot confirm if participants watched assigned videos, completed practice activities, and/or if quantity of participation in asynchronous activities had an impact on study outcomes. As emphasized by Lichstein et al. (33), delivery receipt is an independent treatment component that must be assessed to determine if a valid clinical trial has been conducted. Therefore, future iROLL process evaluations must capture participants' receipt of synchronous, as well as asynchronous, content. Considering the usage of application technology to monitor dose received could be a viable option. The process evaluation strategies utilized in the future can also be strengthened by applying best practices in survey research. Specifically, double or multi-barreled questions (e.g., on the iROLL-O's *Trainer Fidelity Form*) must be avoided. In addition, although the study utilized feedback from both interventionists and end users, the numbers of people in both stakeholder groups were small. Feedback from larger samples of stakeholder groups would yield more robust data to inform evaluation design and intervention development. Future studies involving larger numbers of participants from a variety of geographic regions are needed to improve generalizability of the findings. Fidelity was also measured by trainer self-report. An external rater of fidelity would increase the validity of the session fidelity findings. Finally, given that findings suggested that participants' community participation, a long-term goal of the iROLL-O intervention, was negatively impacted by the COVID-19 pandemic, enhancing process evaluation strategies to better understand contextual influences on intervention participation and outcomes is warranted.

## Conclusion

Feedback from key stakeholders was essential to an informative process evaluation. Online delivery supported program implementation, including reach, and resulted in high levels of satisfaction among participants and trainers. Individualized Reduction of FaLLs content and processes that apply social learning theory and application of self-management strategies were closely tied to MOC and were supported by online program delivery by skilled occupational and physical therapy interventionists. Future iterations should aim to uphold the positive group context, recruit, and train licensed occupational or physical therapists as interventionists, and continue to provide the program's diverse approach to fall prevention and management. Revisions to enhance participants' technical capabilities and relatability of program materials are indicated.

## Data availability statement

The raw data supporting the conclusions of this article will be made available by the authors, without undue reservation.

## Ethics statement

The studies involving human participants were reviewed and approved by the study was approved by the Human Research Protection Offices at the three collaborating sites: the University of Illinois Chicago (UIC), the University of Illinois Urbana-Champaign (UIUC), and the Shepherd Center (SC) in Atlanta, GA. The patients/participants provided their written informed consent to participate in this study.

## Author contributions

TV: conceptualization and design, data analysis and interpretation, methodology, manuscript drafting, and revision. EP and LR: conceptualization and design, data analysis and interpretation, methodology, manuscript drafting and revision, funding acquisition, and supervision. AM, RY, SS, JK, and AS: data analysis and interpretation, methodology, manuscript review, and revision. All authors contributed to the article and approved the submitted version.
